# Human Cord Blood Derived Immature Basophils Show Dual Characteristics, Expressing Both Basophil and Eosinophil Associated Proteins

**DOI:** 10.1371/journal.pone.0048308

**Published:** 2012-10-31

**Authors:** Jeanette Grundström, Jenny M. Reimer, Sofia E. Magnusson, Gunnar Nilsson, Sara Wernersson, Lars Hellman

**Affiliations:** 1 Department of Cell and Molecular Biology, Uppsala University, Uppsala, Sweden; 2 Clinical Immunology and Allergy Unit, Department of Medicine, Karolinska Institute, Stockholm, Sweden; 3 Isconova AB, Uppsala, Sweden; 4 Department of Anatomy, Physiology and Biochemistry, Swedish University of Agricultural Sciences, Uppsala, Sweden; Université Libre de Bruxelles, Belgium

## Abstract

Basophils are blood cells of low abundance associated with allergy, inflammation and parasite infections. To study the transcriptome of mature circulating basophils cells were purified from buffy coats by density gradient centrifugations and two-step magnetic cell sorting. However, after extensive analysis the cells were found to be transcriptionally inactive and almost completely lack functional mRNA. In order to obtain transcriptionally active immature basophils for analysis of their transcriptome, umbilical cord blood cells were therefore cultured in the presence of interleukin (IL)-3 for 9 days and basophils were enriched by removing non-basophils using magnetic cell sorting. The majority of purified cells demonstrated typical metachromatic staining with Alcian blue dye (95%) and expression of surface markers FcεRI and CD203c, indicating a pure population of cells with basophil-like phenotype. mRNA was extracted from these cells and used to construct a cDNA library with approximately 600 000 independent clones. This library served as tool to determine the mRNA frequencies for a number of hematopoietic marker proteins. It was shown that these cells express basophil/mast cell-specific transcripts, i.e. β-tryptase, serglycin and FcεRI α-chain, to a relatively low degree. In contrast, the library contained a high number of several eosinophil-associated transcripts such as: major basic protein (MBP), charcot leyden crystal (CLC), eosinophil cationic protein (ECP), eosinophil derived neurotoxin (EDN) and eosinophil peroxidase (EPO). Out of these transcripts, MBP and EPO were the most frequently observed, representing 8% and 3.2% of the total mRNA pool, respectively. Moreover, in a proteome analysis of cultured basophils we identified MBP and EPO as the two most prominent protein bands, suggesting a good correlation between protein and mRNA analyses of these cells. The mixed phenotype observed for these cells strengthens the conclusion that eosinophils and basophils are closely linked during human hematopoietic development. The dual phenotype also indicates that other cytokines than IL-3 or cell surface interactions are needed to obtain the full basophil specific phenotype *in vivo*.

## Introduction

Basophils have historically often been described as blood mast cells (MCs) since they both stain positive for basic dyes and contain histamine. However, despite large similarities in humans they clearly represent two separate cell lineages. Basophils normally constitute less than 1% of the circulating leukocytes. In contrast, MCs are much more common, are rarely found in peripheral blood and are essentially resident tissue cells [1].

The basophilic granulocyte was first described by Paul Ehrlich in his work ¨Beiträge zur Kenntnis der granulierten Bindegewellzellen und der eosinophilien Leukocyten in the late 19^th^ century [2]. They were given their name due to the fact that they stain with basic dyes like Toluidine Blue and Alcian blue. Basophils are primarily known for their central role in IgE-mediated allergies. However, recently basophils have also attracted major interest as a potential key initiator for the induction of T-helper type 2 (Th2) immune responses to invading pathogens. Supporting evidence for this Th2 priming function of the basophil comes from studies demonstrating that basophils express and release substantial amounts of IL-4, IL-13 and thymic stromal lymphopoietin (TSLP) [3], [4], [5], [6], [7], [8], [9], [10]. In favor of a major importance for the cytokines produced by basophils is the fact that the mouse MC protease (mMCP)-8 positive basophils identified during malaria infection produce up to 40 times more IL-4 than fully activated Th2 cells [5]. Basophils have also been identified as the most important source of IL-4 during the first hours after antigen challenge [7]. IL-4 and IL-13 are both key cytokines in Th2 mediated immune responses and these are the only two cytokines known to induce B cells to switch to IgE [11]. A number of studies have recently also shown the importance of basophils in various inflammatory conditions. They have been shown to play a pivotal role in the induction of eosinophil infiltration during the induction phase of atopic dermatitis [12]. They are also essential for IgG but not IgE mediated anaphylaxis in mice [13]. Interestingly, basophils have been shown to be of major importance for the memory response to ectoparasites, like ticks [14]. From numerous studies the general view has emerged that basophils and mast cells have distinct roles in allergy and asthma, as reviewed in [15], and that basophils are involved in the defense against both endo- and ecto-parasites [16], [17], [18].

Although basophils have been known for more than 100 years, very little is known about their granule content, especially for human basophils. It has been shown that human basophils store small amounts of the mast cell tryptase (Blom and Hellman unpublished results) and [19], whereas human MCs store large amounts of proteases, belonging to both the tryptase and the chymase families [20], [21]. In contrast to human basophils two basophil specific proteases have been described in the mouse, mMCP-8 and mMCP-11, and mouse basophils do not express any of the MC associated serine proteases, mMCP-1, −2, −4, −5, −6- or −7 [5], [22], [23], [24]. However, no basophil specific human protein has yet been cloned and no homologue to mMCP-8 can be identified in the human genome [25], [26]. Interestingly, the mMCP-8 gene including its regulatory elements has recently been used to specifically ablate basophils showing the specificity of this protein marker to murine basophils [14], [16], [27]. Two human basophils specific proteins have been identified basogranulin and a protein reacting against monoclonal 2D7 [28], [29]. However, none of these have been characterized in more detail or cloned, in spite of massive efforts.

A major obstacle in the identification of new basophil-specific proteins has been the difficulty in obtaining sufficient numbers of pure basophils in combination with an almost complete absence of functional mRNA in peripheral blood basophils. The explanation for the extremely low mRNA levels is most likely that, like certain other leukocytes, i.e. neutrophils and eosinophils, basophils mature in the bone marrow and enters the circulation as terminally differentiated cells with very little ongoing protein synthesis. The cloning of novel lineage-specific genes from other granulocytes have therefore almost exclusively come from the use of immortalized tumor cell lines [30] or *in vitro*-cultivated cells [31]. These cells are actively dividing and contain large amounts of mRNA. Two tumor cell lines with basophil origin have also been described, KU812 and LAMA84 [32], [33], [34]. Both of these cell lines do however display characteristics of multiple lineages, suggesting that they are not optimal for studies of lineage-specific granule proteins. An alternative is therefore to use cultured basophil precursors. It is well established that IL-3, in human cord-blood or bone marrow cultures, promotes the expansion of a cell type closely resembling basophils as demonstrated by cell surface expression, staining properties and functional characteristics [35], [36], [37].

In order to obtain immature transcriptionally active basophils for an analysis of their transcriptome and to clone human basophil specific proteins a project was initiated to study conditions needed to obtain immature basophils in sufficient amounts to construct a high quality cDNA library. Such culture conditions were optimized and we managed to obtain preparations of more than 34 million immature basophils for mRNA purification and cDNA library construction [38]. These cells showed a number of known characteristics of immature basophils, including metachromatic staining and surface expression of the high affinity receptor for IgE (FcεRI) and CD203c [38]. CD203c has been shown to be a common surface marker for human basophils, mast cells and their CD34^+^ progenitors [39]. CD203c has also been found to be up-regulated upon activation of basophils (as reviewed in [40]).

A cDNA library from a preparation of these cells were produced and used as a starting material for a transcriptome analysis. Upon sequence analysis of a panel of randomly picked clones from this library we found that eosinophil related transcripts were present in relatively high numbers, indicating that these cells have a mixed phenotype, displaying both basophil- and eosinophil-related characteristics [38]. We here present a more detailed characterization of this library using a more extensive sequence analysis of a larger number of randomly picked clones and a screening with specific probes. These experiments were performed to give a more detailed picture of the phenotype of IL-3 induced cord blood derived basophils cells as well as an evaluation of their usefulness in studying basophil biology.

## Materials and Methods

### Purification of Blood Basophils

Buffy coats were obtained from the University Hospital in Uppsala. Cells were layered onto Ficoll Hypaque® (GE Healthcare, Uppsala, Sweden) and purified by gradient centrifugation, according to the manufacturer’s instructions. The retrieved cells were then purified by a combination of negative and positive selection by magnetic-activated cell sorting (MACS) (Miltenyi Biotec, Bergisch-Gladbach, Germany). During negative selection a 2-step cocktail procedure was used. Washed cells were primary labeled by adding monoclonal anti-CD4, −CD8, −CD3, −CD14, −CD16 and −CD19 antibodies. Cells were then washed in MACS-buffer (PBS, 2 mM EDTA, 0.5% BSA) and labeled with anti-mouse IgG1 Microbeads (Miltenyi Biotec). Antibody binding was performed on ice for 30 min with gentle shaking. Cells were magnetically separated according to the manufacturer’s instructions. The cells were then subjected at a positive selection step. Cells were first incubated with a mouse IgG1 anti-IgE antibody followed by staining with anti-mouse IgG Microbeads (Miltenyi Biotec). Antibody binding was performed on ice for 30 min with gentle shaking. Cells were then magnetically separated according to the manufacturer’s instructions. The basophils obtained had a purity of 98%, as determined by Alcian blue staining.

### Culture of Umbilical Cord Blood Basophils

After informed consent and after approval from the local Ethical Review Board at the University Hospital, Uppsala, umbilical cord blood was collected from normal, full-term deliveries. Mononuclear cells were isolated from heparinized cord blood by Ficoll Hypaque® (GE Healthcare, Uppsala, Sweden) gradient centrifugation, according to the manufacturer’s instructions. Cells were washed in PBS and re-suspended at a concentration of 1×10^6^ cells/ml in RPMI 1640 medium supplemented with 10% fetal calf serum, 50 µM 2-mercaptoethanol, 10 mM Hepes, 2 mM L-glutamine, 0.1 mM non-essential amino acids, 100 IU/ml penicillin, and 50 µg/ml streptomycin (Life Technologies, Renfrewshire, Scotland). In addition, the medium was supplemented with 10 ng/ml recombinant IL-3 (R&D Systems, Abingdon, UK). The cells were maintained at 37°C and 5% CO_2_ for 9 days. The medium was changed after the first seven days. In order to obtain sufficient material for the construction of the cDNA library and for gel analysis of the proteome we used cells from five different donors. For the cDNA library pooled cells from four donors were used, with 2.1, 16.5, 10.7 and 4.3×10^6^ immature basophils generated from the respective cultures. For SDS-PAGE analysis 1.4×10^6^ cultured immature basophils were isolated from the culture of one donor.

### Histological Staining

Cord blood cells or purified basophils were cytocentrifuged onto slides and analyzed for metachromatic staining of granules by incubating slides in 1% Alcian blue 8 GX (Sigma, St Louis, MO, USA), diluted in 60% ethanol and 3.7% hydrochloric acid, for 15 min. To stain cell nuclei, slides were incubated for 5 min with nuclear fast red counter stain (Apoteksbolaget, Södersjukhuset, Stockholm, Sweden). After staining, slides were rinsed for 2 s in dH_2_0, air-dried and mounted.

### Flow Cytometry

Purification of basophils and analyses of surface marker expression were performed as previously described [38].

### Isolation of Cultured Basophils

Cultured basophils were purified by magnetic-activated cell sorting (MACS) using a 2-step cocktail procedure. Washed cells were primary labeled by adding (per 10^7^ cells): 5 µl FcR-Blocking Reagent (Miltenyi Biotec, Bergisch-Gladbach, Germany), 20 µl IgG1 anti-CD3 (0.1 µg/µl, DAKO A/S, Glostrup Denmark), 10 µl IgG1 anti-CD16 (0.1 µg/µl, DAKO A/S), 20 µl IgG1 anti-CD36 (0.04 µg/µl, Immunotech, Marseille, France), and 35 µl IgG1 anti-CD45RA (0.05 µg/µl, Immunotech). Cells were then washed in MACS-buffer (PBS, 2 mM EDTA, 0.5% BSA) and labeled in 100 µl/10^7^ cells with 20 µl anti-mouse IgG1, 10 µl anti-CD14, and 10 µl anti-CD15 Microbeads (Miltenyi Biotec). Staining was performed on ice for 30 min with gentle shaking. MACS-buffer corresponding to 20 times the secondary staining volume was added before separation. Cells were magnetically separated on CS Columns according to the manufacturer’s instructions.

### Construction of a cDNA Library

Total RNA was isolated from the purified basophils by the guanidine thiocyanate method [41] and poly(A)^+^ RNA was isolated using the PolyATtract®I system (Promega, 2800 Woods Hollow Road, Madison, WI). Subsequently, double stranded cDNA was synthesized using the TimeSaver™ cDNA Synthesis Kit (Amersham Pharmacia Biotech, Uppsala, Sweden). The cDNA was then ligated into the single *Eco*RI site of the lambda gt-10 vector (Promega) and packaged using the Packagene® Lambda DNA Packaging System (Promega). The library from the blood basophils and the cultured basophils were found to contain approximately 14 000 and 600 000 independent recombinants, respectively.

### Screening and Sequencing of Randomly Picked Clones

Seven hundred clones from the cultured immature basophils were randomly picked, and the insert fragments were amplified with vector specific primers. After purification the insert DNA fragments were sent for sequencing at Uppsala Genome Center. The sequences were screened against the entire human genome by Blast search of the NCBI database.

### Analyses of Transcript Frequencies with Specific Probes

Approximately 130 000 plaques from the unamplified cDNA library were spread as a monolayer of the *E-coli* C600 Hfl strain with a titer of approximately 25 000 plaques/plate on eight plates. The plaques were transferred to Hybond N+ filters (Amersham Int., Amersham, Buckinghamshire, England). Purified cDNA fragments were labeled with ^32^P by random priming and used as probes (Megaprime, Amersham Int.). The filters were screened with the various cDNA and oligo probes. The filters were washed at high stringency (0.1×SSC, 0.1% SDS) for cDNA probes and at medium stringency (2×SSC, 0.5% SDS) for oligo nucleotide probes. Autoradiography was performed for 24–48 h on Kodak Exomat AR film (Eastman-Kodak Company, Rochester New York, USA).

### SDS-PAGE Analysis

Approximately 1 million cord blood derived immature basophils were dissolved in SDS-sample buffer and separated on a 13% SDS-PAGE gel. As a reference we used the same number of cells from a human embryonic kidney cell line HEK 293-EBNA. The gel was then stained with Coomassie brilliant blue.

### Mass Spectroscopy

Following SDS-PAGE analysis and Coomassie brilliant blue staining gel bands were excised and the protein was eluted and digested with trypsin. The peptides generated after trypsin cleavage was then analyzed by mass spectroscopy. The detailed molecular weight information from these fragments was then matched against the NCBI database of peptide fragments from the entire human genome. This resulted in a few potential proteins, usually 2–7. Analysis of several of the generated fragments from a single gel band resulted in only one protein that matched all peptides. Typically only two or three peptides were needed to obtain a single target protein. However, at least five peptides were always analyzed to obtain a highly reliable determination.

## Results

### Isolation of Peripheral Blood Basophils

To study the transcriptome of basophils we initially used peripheral blood basophils in a series of experiments. Buffy coats were used as starting material to obtain sufficient numbers of human blood basophils for mRNA analysis and cDNA library construction. The cells were purified on Ficoll Hypaque and by magnetic cell sorting. In the last of these experiments, where we performed the most extensive analysis, we used 20 million basophils of a purity of 98%, as determined by Alcian blue staining. Due to the very small amounts of nucleic acids in the preparation we used the entire material to construct a cDNA library for transcriptome analysis. A cDNA library with a total of 14 000 clones was obtained. These clones were plated on two plates and plaque lifts to two hybond N+ filters were performed which were then hybridized with ^32^P-labelled probes against β-actin, the mast cell tryptase, the IgE high affinity receptor α-chain, and a mononuclear cell cDNA probe. A few very weak signals were obtained with these probes. Phage DNA from these clones were purified and analyzed by restriction enzyme digestion. All of these clones contained very short insert DNA fragments. Sequence analysis of the inserts revealed that all clones contained Alu repeats or other chromosomal DNA fragments. No single clone originating from an mRNA was identified. These results indicated that peripheral blood basophils contain very low levels of intact mRNA. A few additional basophil preparations were also analyzed with very similar results, no intact mRNA. A likely explanation to these results is that peripheral blood basophils are terminally differentiated and contain almost no functional mRNA. Based on these results we decided to focus our effort to study the basophil transcriptome on *in vitro* differentiated basophils as described below.

### Isolation of Immature Basophils from Cord Blood Cell Cultures

**Figure 1:**
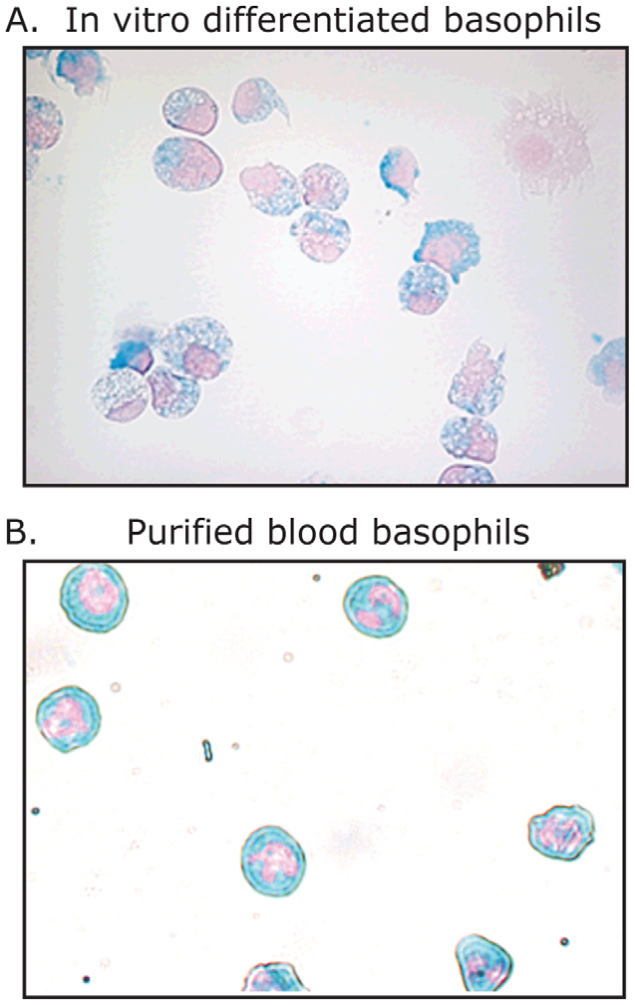
Alcian blue staing of *in vitro* differentiated basophils and normal blood basophils. Panel A shows Alcian blue stained cells from day 9 of *in vitro* differentiated basophils originating from umbilical cord blood cultures stimulated with a continuous presence of 10 ng/ml IL-3. Panel B shows Alcian blue stained normal purified peripheral blood basophils.

To obtain transcriptionally active basophils in sufficient numbers for an analysis of their trascriptome, mononuclear cells were isolated from umbilical cord blood. Cells were cultured in the continuous presence of IL-3 for nine days (10 ng/ml). After nine days in culture approximately 60% of the cells were basophil-like as determined by metachromatic staining. Using magnetic cell sorting and a 2 step cocktail 34×10^6^ immature basophil-like cells were isolated. This protocol resulted in both high purity (95%±0.5%, Alcian blue-positive) and a recovery of (59%±1.5%) of the cultured basophils (Fig 1A). Of the purified cells 76% expressed the mast cell and basophil marker CD203c and 56% expressed the high affinity IgE receptor, FcεRI [38]. The phenotype of the cells were similar to human blood basophils, however, with slightly less bright blue staining and with a round nuclei, and not the lobulated nuclei of the blood basophils (Fig 1B). A detailed description of the phenotype of these cells, including FACS figures, is presented in a previous publication [38].

### Construction and Screening of a cDNA Library from Immature Basophils

mRNA was isolated, from the 34×10^6^ immature basophils described above by oligo dT selection. The mRNA was used to construct a cDNA library in the phage λ gt10 vector with approximately 600 000 independent recombinants. Approximately 700 independent clones were isolated from this library and analyzed for insert sizes by PCR with vector specific primers. Approximately 40% of the clones (296 clones), contained inserts with a size of more than 100 bp. The nucleotide sequence of these inserts were determined and compared against the human genome database (NCBI). Approximately 55% of these clones contained identifiable cDNA inserts (173 sequences). The rest of the clones were chromosomal DNA fragments, possibly originating from apoptotic cells. A complete list of the 173 inserts, which were found to originate from a broad range of different genes, is found in table 1. A majority of the inserts (70%) are from housekeeping genes and 16% from lineage-specific genes. Almost all of the housekeeping genes were found only once among these 173 clones. However, most of the lineage specific genes were found as several clones, demonstrating the overrepresentation of lineage-specific transcripts in the library. Eighteen of the isolated clones encoded the major basic protein (MBP), two encoded charcot leyden protein (CLC) and two encoded a MBP homologue (Table 1). These three genes are expressed by both eosinophils and basophils. Three clones encoded the eosinophil specific protein eosinophil peroxidase (EPO), and two clones encoded the serglycin proteoglycan core protein, which is expressed by several hematopoietic lineages (Table 1). We could however not find any clones for the high affinity IgE receptor subunits or for the mast cell tryptase. This prompted us to screen the library for additional lineage-specific transcripts by other more sensitive techniques, than by direct sequencing.

**Table 1:** Clones isolated from the basophil-like cell cDNA library.

Name of protein	Genbank accession number	No. of sequences
**Lineage specific proteins**		
Major basic protein (MBP, proteoglycan 2)	BC005929, Y00809, NM_002728	18
Charcot Leyden–crystal protein (CLC)	L01664	2
Proteoglycan 1	X17042	2
MBP-homologue, proteoglycan 3	NM_006093	2
Leukocyte associated Ig like receptor 1	NM_021706	1
Eosinophil peroxidase (EPO)	X14346	3
**Cell structural genes**		
Tubulin β	BC001002, AB062393	2
Actin β	BC004251	1
Actin γ1	BC039144	1
**Enzymes**		
Acylphosphatase 2	BC012290	1
Aldolase A	BC010660	1
Aminolevulinate δ synthase 1	BC011798	1
Asparagine-linked glycosylation 8 homologue	BC001133	1
ATP-ase lyzosomal accessory protein 1	BC000724	1
Carnithine O-octanoyl transpherase	BC051874	1
Cyclin dependent kinase regulatory subunit 2	BC006458	1
Diacylglycerol kinase zeta	AK123378	1
Disulfide isomerase	AY358646	1
Enolase 1	NM_080738, NM_001428	2
Esterase D	AF112219	1
DEAD (Asp-Glu-Ala-Asp) box polypeptide 3	NM_001356	1
DEAD (Asp-Glu-Ala-Asp) box polypeptide 27	BC016060	1
Flap structure specific endonuclease 1	NM_004111	1
Glia maturation factor γ	BC032819	1
Glucosaminyl transferase 1	NM_001490	1
Glutathione-S-transpherase zeta	BC001453	1
IMP hydrolase	BC008879	1
KRIT 1	AF388384	1
Lactate dehydrogenase B	BC002362	1
Methylthioadenosine phosphorylase	BC026106	1
NADH dehydrogenase 1 α subcomplex 9	BC009311	1
Ornithine decarboxylase 1	NM_002539	1
Phosphatidic acid phosphatase type 2A	BC039847	1
Phosphoantothenate cysteine ligase	BC062586	1
Phosphoribosylaminoimidazole carboxylase	BC010273	1
PI-3 kinase related kinase SMG-1	BC061522	1
Prolyl 4-hydroxylase α subunit	M24487	1
Protin kinase, DNA activated, catalytic polypep.	NM_006904	1
SP25	D14658	1
Transketolase	BC024926	1
Translation initiator factor 2α kinase 3	NM_004836	1
Triosephosphatase isomerase 1	BC009329	1
**Genes of the immune system**		
MHC-1, HLA-Bw62	M28204	1
CD74	NM_004355	1
β2−microglobulin	BC032589	1
Translationally controlled tumor protein 1	BC052333	3
Proteosome activator subunit 3	NM_005789	1
T1A1 cytotoxic granule associated RNA binding protein	NM_022173	1
Oxidation resistance 1	BC032710	1
Peroxiredoxin 2	BC000452	1
**Genes involved in lipid metabolism**		
Oxysterol binding protein	NM_002556	1
**Genes involved in the protein translation machinery**		
Ribosomal protein L3	BC012786	1
Ribosomal protein L7	NM_000971	1
Ribosomal protein L7a	NM_000972	2
Ribosomal protein L12	NM_000976, BC050644	2
Ribosomal protein L13a	NM_012423	1
Ribosomal protein L15	NM_002948	1
Ribosomal protein L18a	NM_000980	1
Ribosomal protein L23a	BC014459, BC058041	2
Ribosomal protein L26	NM_000987	1
Ribosomal protein S3	NM_001005	1
Ribosomal protein S8	NM_001012	1
Ribosomal protein S10	BC001032	1
Ribosomal protein S11	BC007945	1
Ribosomal protein S16	BC007977	1
Ribosomal protein S18	NM_022551	1
Ribosomal protein S25	NM_001028	1
Ribosomal protein S27	BC002658	1
Ribosomal protein large P0	NM_001002	1
Translation initiation factor 4A	BC012547	1
Translation elongation factor 1α1	BC018641	3
Translation elongation factor 1β2	NM_001959	1
Translation elongation factor 1γ	BC019051	1
RNA polymerase III	NM_007055	1
**Growth and differentiation related proteins**		
Cyclin B1	NM_031966	1
Structural maintenance of chromosome 1 like 1	NM_006306	1
Lectin galactoside binding, soluble 1	BC020675	1
**Regulatory proteins**		
F-box only protein 9	NM_033481	1
GM2 activator protein	X62078	1
**Ion channels, sorting proteins and transporters**		
Facilitated glucose transporter, member 6	BC013740	1
Soluble carrier organic anion transporter member 3A1	AF205074	1
Vacuolar protein sorting 29	NM_016226	1
Sorting nexin 11	NM_013323	1
B-cell receptor associated protein 31	BC014323	1
Ferritin light polypeptide	BC004245	1
Chloride intracellular channel 1	NM_001288	1
Epsin 4	BC004467	1
**Mitochondrial genes**		
Mitochondrial ribosomal protein S15	NM_031280	1
ATP-synthase, H+ transporter, mitochondrial F1 complex, β polypeptide	BC016512	1
Solute carrier family 25, member 5	NM_001152	1
		1
**Proteins associated with cytoskeleton**		
Thymosin β4, X-linked	NM_021109	3
Thymosin β10	BC016731	1
Diaphanous homologue 1	NM_005219	1
Cofilin 1	BC012265	1
Transgelin 2	BC009357	1
Restin	AF143235	1
**Proteins involved with RNA**		
RNA binding motif protein 3	BC006825	1
RNA binding motif protein 6	BC046643	1
Calcium homeostasis, ER protein	BC021294	1
Splicing factor prp8	AF092565	1
Heterogeneous nuclear nucleoprotein A1	NM_002136	1
Heterogeneous nuclear nucleoprotein C	BC003394	1
Cancer susceptibility candidate 3	BC050526	1
Cleavage stimulation factor	M85085	1
**Signal proteins**		
Adenylate cyclase-associated protein 1	BC013963	1
Membrane protein palmitoylated 1, 55 kDa	NM_002436	1
Guanine nucleotide binding protein	BC019093	1
Jagged 1	NM_000214	1
**Stress induced proteins and chaperones**		
Heat shock protein 70 kDa protein 5	BC020235	1
Heat shock protein 70 kDa protein 8	BC008907, BC019816	3
GRP78	AJ271729	1
T complex protein 1, δ subunit	AF026291	1
Cyclophilin B	BC032138	1
**Transcription factors and other DNA binding proteins**		
Zinc finger protein 9	BC014911	1
Zinc finger protein 70	NM_021916	1
Zinc finger protein 207	BC000962	1
General transcription factor IIIC	BC060821	1
Transcription factor Dp-2	BC013993	1
c-myc transcription factor	L16785	1
Hematopoietically expressed homeobox	NM_002729	1
High mobility group nucleosomal binding domain 2	NM_005517	1
**Hypothetical and other proteins**		
Hypothetical protein MGC13204	BC005106	1
Hypothetical protein MGC14156	BC007876	1
Hypothetical protein MGC17943	BC020522	1
Hypothetical protein MGC46719	BC035727	1
Hypothetical protein DKFZP4341216	BC054486	1
Hypothetical protein KIAA0556	XM_044632	1
Hypothetical protein FLJ10719	NM_018193	1
Hypothetical protein FLJ11171	BC035005	1
Hypothetical protein LOC170371	XM_378226	1
Hypothetical protein LOC389865	XM_374329	1
Sushi domain containing	NM_022486	1
GPP34 realted protein	NM_018178	1
Ral-GDS related protein	NM_153615	1
Tetratricopeptiderepeat domain11	BC009428	1

The sequences of the individual clones were screened against the BLASTn database.

### Analyses of Lineage Specific Genes in the Basophil Library

In order to determine the levels of lineage-specific genes present at low numbers, a set of filters were prepared and hybridized with P^32^ labeled cDNAs or oligonuclotide probes directed against a number of hematopoietic marker genes. We analyzed the expression of two basophil and MC related genes, the β-tryptase and the FcεRI α chain, two neutrophil expressed genes, cathepsin G and neutrophil elastase, three eosinophil expressed genes, eosinophil peroxidase (EPO), eosinophil cationic protein (ECP), eosinophil derived neurotoxin (EDN), and two genes that are expressed by both eosinophils and basophils, charcot leyden crystal protein (CLC) and major basic protein (MBP). As a reference we also measured the levels of cytoplasmic β-actin. Although positive signals were detected for all of these probes the expression levels differed substantially between these genes (Table 2). A striking result from this screening was the very high numbers of clones observed for several eosinophil related proteins, exemplified by MBP (500 clones/plate) and EPO (200 clones/plate), which by far exceeded the β-actin levels (120 clones/plate). This should be compared with the relatively low levels of the basophil and neutrophil related genes, resulting in only 2–7 clones/plate for β-tryptase, FcεRI, cathepsin G and N-elastase. The result from this screening thereby shows that the cells in culture, which by staining criteria and surface markers closely resembles basophils are actually more ¨eosinophil-likë when looking at their transcriptome.

**Table 2:** Expression levels of lineage specific genes.

Probe	Signals/filter (average)	Percent of total mRNA pool
Human β-tryptase	2	0.03%
Human FcεRI α-chain	3	0.05%
Human Cathepsin G	7	0.11%
Human N-elastase	2	0.03%
Human MBP	500	8.00%
Human EPO	200	3.20%
Human CLC	150	2.40%
Human EDN	40	0.64%
Human ECP	35	0.56%
Mouse β-actin	120	1.92%

Screening of the human immature basophil library with probes for a panel of different hematopoietic lineage specific markers. Cytoplasmic β-actin was used as a reference. Each filter contained approximately 25 000 plaques. Approximately 25% of the clones in the cDNA library contained identifiable cDNA sequences. Major basic protein (MBP), charcot leyden crystal (CLC), eosinophil cationic protein (ECP), eosinophil derived neurotoxin (EDN) and eosinophil peroxidase (EPO).

### SDS-PAGE Analysis of the Proteome

**Figure 2:**
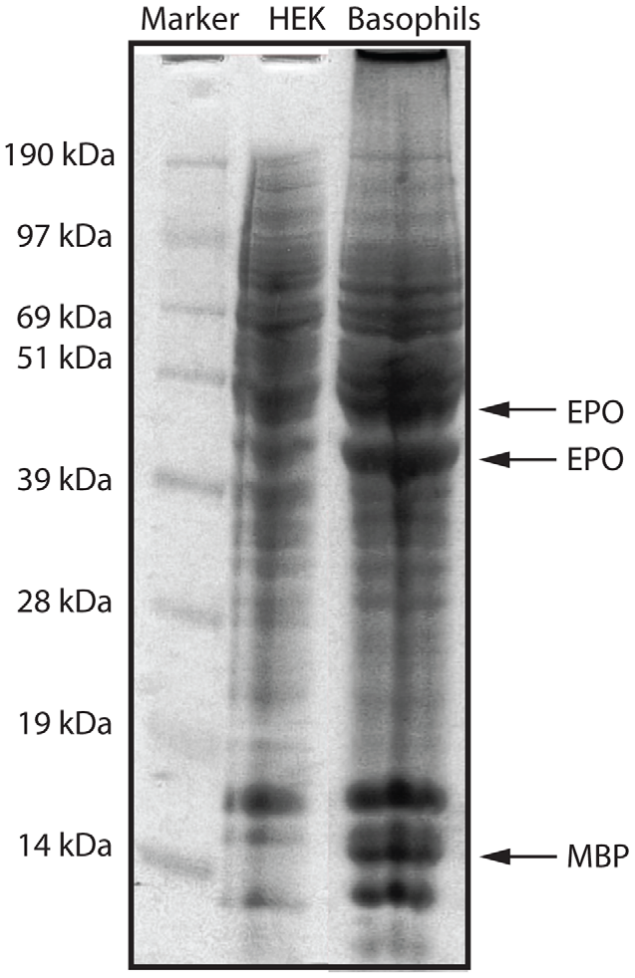
Analysis of the proteome of *in vitro* differentiated basophils. SDS-PAGE analysis of human *in vitro* differentiated basophils. Approximately 1 million cells were dissolved in SDS-sample buffer and separated on a 13% SDS PAGE gel. As a reference the same number of cells from a human embryonic kidney cell line HEK 293-EBNA was used. The positions of three protein bands that were excised from the gel and analyzed by tryptic digestion and mass spectroscopy analysis are indicated by arrows and marked with the proteins identified in these bands: eosinophil peroxidase (EPO) and major basic protein (MBP).

In order to study the proteome of the *in vitro* differentiated basophils cells additional 9-day cultures were analyzed by SDS-PAGE. When comparing the bands against HEK 293 cells, a human embryonic kidney cell line, we could detect a few prominent additional bands in the basophils that were not present in the HEK 293 cells (Fig 2). Several of these additional bands were excised from the gel and subjected to trypsin digestion and mass spectroscopy analysis. The individual peptide fragments generated by trypsin were compared to peptides from the entire human proteome. Two of the bands on the gel resulted in peptide fragments that fully matched with human EPO and MBP, respectively. These were the only two proteins we could identify from the gel with high certainty. EPO and MBP were also the two proteins for which we obtained the highest number of clones in the transcriptome analysis, thus demonstrating that there was a good correlation between protein and mRNA analyses of the cultured basophils.

## Discussion

The aim of this study was to characterize the immature basophil-like cells that were generated during *in vitro* culture of cord blood cells, and to evaluate them as a potential model for studies of basophil biology.

The screening of different culture conditions had previously shown that there is an advantage of culturing cells in the constant presence of IL-3 as compared to pulsing with IL-3, if one wants large amounts of immature transcriptionally active basophils [38]. In order to obtain sufficient numbers of immature basophils for transcriptome analysis we therefore decided to use the protocol involving continuous presence of IL-3 and to culture them for nine days [38].

Using these culture condition and a two step MACS separation protocol we obtained sufficient numbers of 95% Alcian blue positive and transcriptionally active basophil like cells for the production of a cDNA library with 600 000 independent clones. When analyzing the clones in this library we observed that 40% contained inserts of a size of more than 100 bp and that a substantial fraction of these clones contained chromosomal DNA fragments. The latter result indicated that a fraction of the cells already were terminally differentiated and starting to undergo apoptosis. As a result of the high amount of apoptotic DNA the percentage of clones with mRNA copies was somewhere between 20 and 25%. This number was however sufficient to get a relatively detailed picture of the transcriptome of these *in vitro* differentiated basophils. This should be compared with our repeated experiments with normal blood basophils where almost 100% of the clones with inserts contained chromosomal DNA and it was difficult to isolate even a single insert originating from mRNA.

Based on histochemical staining and cell surface expression we characterized the cultured cells as basophil-like. These cells stain with Alcian blue, a characteristic only seen from human peripheral blood basophils and tissue MCs (Fig 1). The majority of the cells in the culture were also surface positive for the IgE high affinity receptor and the MC/basophil related cell surface protein CD203c. However, when we analyzed the transcriptome they were found to be much more eosinophil- than basophil-like. Very high mRNA levels of EPO, MBP, CLC were detected as well as high levels of ECP and EDN, and the levels of the basophil markers FcεRI and β-tryptase were comparably much lower. For some basophil markers the levels were almost 100 fold lower than for the eosinophil markers. For example, with the EPO probe we detected 200 clones/plate compared to the 2 clones/plate for β-tryptase and 3 clones/plate for FcεRI α chain. This shows that although the cells, by staining and cell surface marker expression, resemble immature basophils they are more eosinophil-like when analyzing the transcriptome. This mixed phenotype further strengthens the old notion that in humans eosinophils and basophils are closely linked during development [42], [43], [44]. Interestingly, this situation does apparently not apply for murine basophils where basophils and mast cells seem to have a common precursor [45], [46], [47]. A mouse cell line with dual characteristics of MC and basophils have also been identified, which express both the MC proteases mMCP-5 and CPA and the basophil specific serine protease mMCP-8 [48].

Based on the early findings that human basophils and eosinophils appears to be closely linked during development we expected to see low levels of eosinophil transcripts in the *in vitro* differentiated basophils. However, the very high levels of eosinophil related proteins are difficult to explain. One potential explanation for the low levels of basophil related transcripts could be that these transcripts increase in abundance during differentiation and that eosinophil differentiation is the dominating developmental program. However, then one expects to see relatively high levels of eosinophil related proteins in blood basophils, which is normally not seen, possibly except MBP that has been found both in basophils and MCs [49]. The most likely explanation may therefore be that an unknown factor is needed to push these immature precursors to a more basophil like phenotype. However, the nature of such a factor is still unknown. It is also possible that cell surface interactions with stromal cells are of importance for this developmental process. Interestingly, injection of recombinant IL-3 in monkeys have been found to result in an increase of circulating basophils from 1% to almost 40%, which shows the importance of IL-3 for basophil proliferation *in vivo* [50]. IL-3 thereby appears to be sufficient to increase the number of circulating basophils *in vivo*, but *in vitro* it seems that additional factors are needed to induce this program. Alternatively, other factors could be present in sufficient amounts *in vivo* to support proper development of basophils when IL-3 is added externally. Interestingly, thymic stromal lymphopoietin (TSLP) has recently been shown to induce basophil development independently of IL-3 in mouse and these basophils are functionally distinct from the basophils that appear after IL-3 stimulation [51]. This shows that other cytokines may be of major importance for basophil development.

In summary, the work on the basophil like cells have resulted in the identification of factors and conditions for basophil differentiation as well as the close connection during differentiation of human basophils and eosinophils. However, based on the transcriptome analysis it can be concluded that the culture conditions needs to be optimized to obtain cells that not only show the surface phenotype of mature blood basophils but also a granule content of normal blood basophils.

## References

[1] Hallgren J, Gurish MF (2011) Mast cell progenitor trafficking and maturation. Adv Exp Med Biol 716: 14–28. 21713649 10.1007/978-1-4419-9533-9_2 PMC3554263

[2] Ehrlich P (1879) Beiträge zur Kenntnis der granulierten Bindegewellzellen und der eosinophilien Leukocyten. Arch Anat Physiol: 166–169.

[3] Arock M, Merle-Beral H, Dugas B, Ouaaz F, Le Goff L, et al (1993) IL-4 release by human leukemic and activated normal basophils. J Immunol 151: 1441–1447. 7687630

[4] Li H, Sim TC, Alam R (1996) IL-13 released by and localized in human basophils. J Immunol 156: 4833–4838. 8648131

[5] Poorafshar M, Helmby H, Troye-Blomberg M, Hellman L (2000) MMCP-8, the first lineage-specific differentiation marker for mouse basophils. Elevated numbers of potent IL-4-producing and MMCP-8-positive cells in spleens of malaria-infected mice. Eur J Immunol 30: 2660–2668. 11009100 10.1002/1521-4141(200009)30:9<2660::AID-IMMU2660>3.0.CO;2-I

[6] Min B, Prout M, Hu-Li J, Zhu J, Jankovic D, et al (2004) Basophils produce IL-4 and accumulate in tissues after infection with a Th2-inducing parasite. J Exp Med 200: 507–517. 15314076 10.1084/jem.20040590 PMC2211939

[7] Khodoun MV, Orekhova T, Potter C, Morris S, Finkelman FD (2004) Basophils initiate IL-4 production during a memory T-dependent response. J Exp Med 200: 857–870. 15466620 10.1084/jem.20040598 PMC2213291

[8] Mitre E, Taylor RT, Kubofcik J, Nutman TB (2004) Parasite antigen-driven basophils are a major source of IL-4 in human filarial infections. J Immunol 172: 2439–2445. 14764715 10.4049/jimmunol.172.4.2439

[9] Schneider E, Thieblemont N, De Moraes ML, Dy M (2010) Basophils: new players in the cytokine network. Eur Cytokine Netw 21: 142–153. 20837449 10.1684/ecn.2010.0197

[10] Sokol CL, Barton GM, Farr AG, Medzhitov R (2008) A mechanism for the initiation of allergen-induced T helper type 2 responses. Nat Immunol 9: 310–318. 18300366 10.1038/ni1558 PMC3888112

[11] Hellman L (2007) Regulation of IgE homeostasis, and the identification of potential targets for therapeutic intervention. Biomed Pharmacother 61: 34–49. 17145160 10.1016/j.biopha.2006.10.001

[12] Obata K, Mukai K, Tsujimura Y, Ishiwata K, Kawano Y, et al (2007) Basophils are essential initiators of a novel type of chronic allergic inflammation. Blood 110: 913–920. 17409268 10.1182/blood-2007-01-068718

[13] Tsujimura Y, Obata K, Mukai K, Shindou H, Yoshida M, et al (2008) Basophils play a pivotal role in immunoglobulin-G-mediated but not immunoglobulin-E-mediated systemic anaphylaxis. Immunity 28: 581–589. 18342553 10.1016/j.immuni.2008.02.008

[14] Wada T, Ishiwata K, Koseki H, Ishikura T, Ugajin T, et al (2010) Selective ablation of basophils in mice reveals their nonredundant role in acquired immunity against ticks. J Clin Invest 120: 2867–2875. 20664169 10.1172/JCI42680 PMC2912199

[15] Karasuyama H, Mukai K, Tsujimura Y, Obata K (2009) Newly discovered roles for basophils: a neglected minority gains new respect. Nat Rev Immunol 9: 9–13. 19039320 10.1038/nri2458

[16] Ohnmacht C, Schwartz C, Panzer M, Schiedewitz I, Naumann R, et al (2010) Basophils orchestrate chronic allergic dermatitis and protective immunity against helminths. Immunity 33: 364–374. 20817571 10.1016/j.immuni.2010.08.011

[17] Ohnmacht C, Voehringer D (2010) Basophils protect against reinfection with hookworms independently of mast cells and memory Th2 cells. J Immunol 184: 344–350. 19955520 10.4049/jimmunol.0901841

[18] Karasuyama H, Wada T, Yoshikawa S, Obata K (2011) Emerging roles of basophils in protective immunity against parasites. Trends Immunol 32: 125–130. 21168364 10.1016/j.it.2010.11.006

[19] Jogie-Brahim S, Min HK, Fukuoka Y, Xia HZ, Schwartz LB (2004) Expression of alpha-tryptase and beta-tryptase by human basophils. J Allergy Clin Immunol 113: 1086–1092. 15208589 10.1016/j.jaci.2004.02.032

[20] Lutzelschwab C, Pejler G, Aveskogh M, Hellman L (1997) Secretory granule proteases in rat mast cells. Cloning of 10 different serine proteases and a carboxypeptidase A from various rat mast cell populations. J Exp Med 185: 13–29. 8996238 10.1084/jem.185.1.13 PMC2196094

[21] Pejler G, Ronnberg E, Waern I, Wernersson S (2010) Mast cell proteases: multifaceted regulators of inflammatory disease. Blood 115: 4981–4990. 20233968 10.1182/blood-2010-01-257287

[22] Lutzelschwab C, Huang MR, Kullberg MC, Aveskogh M, Hellman L (1998) Characterization of mouse mast cell protease-8, the first member of a novel subfamily of mouse mast cell serine proteases, distinct from both the classical chymases and tryptases. Eur J Immunol 28: 1022–1033. 9541598 10.1002/(SICI)1521-4141(199803)28:03<1022::AID-IMMU1022>3.0.CO;2-1

[23] Charles N, Watford WT, Ramos HL, Hellman L, Oettgen HC, et al (2009) Lyn kinase controls basophil GATA-3 transcription factor expression and induction of Th2 cell differentiation. Immunity 30: 533–543. 19362019 10.1016/j.immuni.2009.02.008 PMC2772996

[24] Ugajin T, Kojima T, Mukai K, Obata K, Kawano Y, et al (2009) Basophils preferentially express mouse Mast Cell Protease 11 among the mast cell tryptase family in contrast to mast cells. J Leukoc Biol 86: 1417–1425. 19703899 10.1189/jlb.0609400

[25] Gallwitz M, Hellman L (2006) Rapid lineage-specific diversification of the mast cell chymase locus during mammalian evolution. Immunogenetics 58: 641–654. 16807746 10.1007/s00251-006-0123-4

[26] Gallwitz M, Reimer JM, Hellman L (2006) Expansion of the mast cell chymase locus over the past 200 million years of mammalian evolution. Immunogenetics 58: 655–669. 16807745 10.1007/s00251-006-0126-1

[27] Lunderius C, Hellman L (2001) Characterization of the gene encoding mouse mast cell protease 8 (mMCP-8), and a comparative analysis of hematopoietic serine protease genes. Immunogenetics 53: 225–232. 11398967 10.1007/s002510100316

[28] McEuen AR, Calafat J, Compton SJ, Easom NJ, Buckley MG, et al (2001) Mass, charge, and subcellular localization of a unique secretory product identified by the basophil-specific antibody BB1. J Allergy Clin Immunol 107: 842–848. 11344351 10.1067/mai.2001.114650

[29] Kepley CL, Craig SS, Schwartz LB (1995) Identification and partial characterization of a unique marker for human basophils. J Immunol 154: 6548–6555. 7759888

[30] Weil SC, Rosner GL, Reid MS, Chisholm RL, Farber NM, et al (1987) cDNA cloning of human myeloperoxidase: decrease in myeloperoxidase mRNA upon induction of HL-60 cells. Proc Natl Acad Sci U S A 84: 2057–2061. 3031662 10.1073/pnas.84.7.2057 PMC304583

[31] Plager DA, Loegering DA, Weiler DA, Checkel JL, Wagner JM, et al (1999) A novel and highly divergent homolog of human eosinophil granule major basic protein. J Biol Chem 274: 14464–14473. 10318872 10.1074/jbc.274.20.14464

[32] Kishi K (1985) A new leukemia cell line with Philadelphia chromosome characterized as basophil precursors. Leuk Res 9: 381–390. 3858609 10.1016/0145-2126(85)90060-8

[33] Blom T, Huang R, Aveskogh M, Nilsson K, Hellman L (1992) Phenotypic characterization of KU812, a cell line identified as an immature human basophilic leukocyte. Eur J Immunol 22: 2025–2032. 1639103 10.1002/eji.1830220811

[34] Blom T, Nilsson G, Sundstrom C, Nilsson K, Hellman L (1996) Characterization of a human basophil-like cell line (LAMA-84). Scand J Immunol 44: 54–61. 8693292 10.1046/j.1365-3083.1996.d01-84.x

[35] Saito H, Hatake K, Dvorak AM, Leiferman KM, Donnenberg AD, et al (1988) Selective differentiation and proliferation of hematopoietic cells induced by recombinant human interleukins. Proc Natl Acad Sci U S A 85: 2288–2292. 3258425 10.1073/pnas.85.7.2288 PMC279976

[36] Valent P, Besemer J, Muhm M, Majdic O, Lechner K, et al (1989) Interleukin 3 activates human blood basophils via high-affinity binding sites. Proc Natl Acad Sci U S A 86: 5542–5546. 2473472 10.1073/pnas.86.14.5542 PMC297659

[37] Kepley CL, Pfeiffer JR, Schwartz LB, Wilson BS, Oliver JM (1998) The identification and characterization of umbilical cord blood-derived human basophils. J Leukoc Biol 64: 474–483. 9766628 10.1002/jlb.64.4.474

[38] Reimer JM, Magnusson S, Juremalm M, Nilsson G, Hellman L, et al (2006) Isolation of transcriptionally active umbilical cord blood-derived basophils expressing FcepsilonRI, HLA-DR and CD203c. Allergy 61: 1063–1070. 16918508 10.1111/j.1398-9995.2006.01149.x

[39] Buhring HJ, Simmons PJ, Pudney M, Muller R, Jarrossay D, et al (1999) The monoclonal antibody 97A6 defines a novel surface antigen expressed on human basophils and their multipotent and unipotent progenitors. Blood 94: 2343–2356. 10498606

[40] Buhring HJ, Streble A, Valent P (2004) The basophil-specific ectoenzyme E-NPP3 (CD203c) as a marker for cell activation and allergy diagnosis. Int Arch Allergy Immunol 133: 317–329. 15031605 10.1159/000077351

[41] Chomczynski P, Sacchi N (1987) Single-step method of RNA isolation by acid guanidinium thiocyanate-phenol-chloroform extraction. Anal Biochem 162: 156–159. 2440339 10.1006/abio.1987.9999

[42] Denburg JA, Telizyn S, Messner H, Lim B, Jamal N, et al (1985) Heterogeneity of human peripheral blood eosinophil-type colonies: evidence for a common basophil-eosinophil progenitor. Blood 66: 312–318. 2410064

[43] Takemori N, Saito N, Tachibana N, Hayashishita N, Miyazaki T (1988) Hybrid eosinophilic-basophilic granulocytes in chronic myeloid leukemia. Am J Clin Pathol 89: 702–703. 3162789 10.1093/ajcp/89.5.702

[44] Boyce JA, Friend D, Matsumoto R, Austen KF, Owen WF (1995) Differentiation in vitro of hybrid eosinophil/basophil granulocytes: autocrine function of an eosinophil developmental intermediate. J Exp Med 182: 49–57. 7540656 10.1084/jem.182.1.49 PMC2192091

[45] Arinobu Y, Iwasaki H, Gurish MF, Mizuno S, Shigematsu H, et al (2005) Developmental checkpoints of the basophil/mast cell lineages in adult murine hematopoiesis. Proc Natl Acad Sci U S A 102: 18105–18110. 16330751 10.1073/pnas.0509148102 PMC1312421

[46] Hallgren J, Gurish MF (2007) Pathways of murine mast cell development and trafficking: tracking the roots and routes of the mast cell. Immunol Rev 217: 8–18. 17498048 10.1111/j.1600-065X.2007.00502.x

[47] Ohmori K, Luo Y, Jia Y, Nishida J, Wang Z, et al (2009) IL-3 induces basophil expansion in vivo by directing granulocyte-monocyte progenitors to differentiate into basophil lineage-restricted progenitors in the bone marrow and by increasing the number of basophil/mast cell progenitors in the spleen. J Immunol 182: 2835–2841. 19234178 10.4049/jimmunol.0802870 PMC2756103

[48] Lunderius C, Xiang Z, Nilsson G, Hellman L (2000) Murine mast cell lines as indicators of early events in mast cell and basophil development. Eur J Immunol 30: 3396–3402. 11093157 10.1002/1521-4141(2000012)30:12<3396::AID-IMMU3396>3.0.CO;2-O

[49] Nakajima T, Matsumoto K, Suto H, Tanaka K, Ebisawa M, et al (2001) Gene expression screening of human mast cells and eosinophils using high-density oligonucleotide probe arrays: abundant expression of major basic protein in mast cells. Blood 98: 1127–1134. 11493461 10.1182/blood.v98.4.1127

[50] Mayer P, Valent P, Schmidt G, Liehl E, Bettelheim P (1989) The in vivo effects of recombinant human interleukin-3: demonstration of basophil differentiation factor, histamine-producing activity, and priming of GM-CSF-responsive progenitors in nonhuman primates. Blood 74: 613–621. 2665851

[51] Siracusa MC, Saenz SA, Hill DA, Kim BS, Headley MB, et al (2011) TSLP promotes interleukin-3-independent basophil haematopoiesis and type 2 inflammation. Nature 477: 229–233. 21841801 10.1038/nature10329 PMC3263308

